# Healthcare Utilisation—Why the Problem of Equalising Access Has Become Even Harder

**DOI:** 10.3390/healthcare11172430

**Published:** 2023-08-30

**Authors:** Sally Fowler Davis

**Affiliations:** Faculty of Health, Medicine and Social Care, Anglia Ruskin University, Cambridge CB1 1PT, UK; sally.fowler-davis@aru.ac.uk

People use healthcare services to diagnose, cure, or ameliorate disease or injury, to improve or maintain function, or to obtain information about their health status and prognosis [1]. Disparities in access to healthcare have been widely recognised to be associated with social and economic deprivation [2,3,4] but are often attributed to information deficits about communities needs at the neighbourhood or population level [5]. In conceptualising access and utilisation, this Special Issue is focused on ageing and the utilisation of the healthcare system based on user entitlement in different healthcare systems, focusing in on the differential access to healthcare. Multimorbidity, social and economic determinants and user expectations are the main considerations for the services. Targeting poorer, older, and more marginalised communities to improve accessibility to the services is now recognised as an important challenge to improve health outcomes internationally. Levesque et al., in 2019 [3], generated five dimensions of accessibility: (1) approachability; (2) acceptability; (3) availability and accommodation; (4) affordability; and (5) appropriateness of services. In addition, they developed a corresponding set of dimensions that identify the relative capabilities of populations to interact with services: (1) ability to perceive; (2) ability to seek; (3) ability to reach; (4) ability to pay; and (5) ability to engage. The connection that they made between provision and engagement usefully highlights the reciprocal nature of the access dilemma and provides a tool for considering the failures to effectively equalise access to all populations.

The World Health Organization states that health is determined by a person’s individual characteristics and behaviours, physical environment, and socioeconomic environment [6]. The environmental context or physical experience of ‘place’ is clearly acknowledged as a fundamental factor in determining a person’s ability to receive care and use services to recover or manage their health. In the UK, an additional factor in healthcare utilisation is the ambition to achieve a net zero NHS by 2030, and thus facilitating new ways of enabling care and access to services without increasing the carbon footprint. As the climate and other environmental factors increasingly impinge on the daily routines of people in their local neighbourhoods and communities, there is a real need to target those individuals and groups identified as more vulnerable. The critical challenge for a service according to Levesque would therefore be to co-produce an understanding of need, relative to place, and to identify the health outcomes that are acceptable and meaningful as health improvements at the population level. To date, our health systems have tended to define the best outcomes according to medical need, but communities will very often define health in a more nuanced way, in terms of household and neighbourhood wellbeing.

For a community, the challenge is to promote health and wellbeing as key assets that lead to valued outcomes. Examples include being sufficiently well to fully participate in education and to be sufficiently well to work and sustain employment. For different groups and populations, health has different meaning and value. Poorer communities, when asked, will sometimes define their health and wellbeing needs in terms of the effects of substandard housing, social cohesion, civic incivilities (i.e., drug crime), and air quality. These concerns are legitimate, being major causes of ill-health and especially mental health conditions. However, the municipal infrastructures (housing, parklands and policing) are rarely within the ability of health services to improve, unless population health is seen as a shared local priority. Perhaps the central challenge for healthcare utilisation is to listen to communities and integrate an understanding of healthcare into the wider infrastructural considerations that include democratic, social, environmental, and economic structures that underpin wellbeing. As communities increasingly diversify, the traditional statutory and regulated healthcare provision needs to take into account social, cultural and economic determinants.

The utilisation of healthcare (including social care) is motivated by multiple factors and the psychological and social need is as important as the more tangible health condition. For example, many older adults on long waiting lists for hip surgery are stoical [7] but grateful when a letter arrives to reassure them that they are still on the waiting list. This is perceived as not having been forgotten. Similarly, young people have been seeking psychological support in huge numbers since the pandemic to reduce their anxieties that manifest as self-harm and disordered eating behaviours. Their behaviours are differentiated across the least and most affluent demographics but demonstrate real psychological distress [8]. In keeping with older adults, they want their needs recognised by a society that has returned to a ‘new normal’ following the COVID-19 pandemic, and their service-seeking reflects this need. The visibility and ease of access to services are factors in these contrasting populations and are an important consideration for those re-designing services. There is an expectation and a clear imperative to understand the demand for healthcare and to work across the health systems to respond and to target need, especially with a universal health offer.

In countries that have universal, free at the point of contact healthcare systems, such as the NHS, there is an advantage, in that the service offer is well understood, at least to those familiar with the system. In countries without a universal offer, there is, perhaps, greater choice for those who access services (especially when they pay), but many confounding factors, which include cost, entitlement, geography, and personal ability, as these moderate the utilisation of health provisions. Where universal health coverage exists, the ‘one-size-fits-all’ service offer is typically based on a medical-centric service design, i.e., surgeries, clinics, and, more recently, tele-care, digital or telephone contact. The fact that there is a widely varied ability to take-up a care offer is weakly understood and there is a need to implement services based on the diversity of needs. Patients’ needs are mostly prioritised according to acute illness (i.e., frailty services and diagnostic cancer services), and whilst the medical urgency is important, the problem remains that equity of access and indeed the consequent outcomes of healthcare are based on demographic factors. It is perhaps important to re-iterate and endorse the impact of the Marmot Reviews [9,10], which completely demonstrate how environmental factors influence how care is sought, offered, and received, what treatments are effective, and who receives treatment. The traditional and often identifiable medically generated patient–practitioner contact has the clear advantage of providing a predictable and often appropriate service model. However, this model is only helpful for the motivated and those able to identify their own health needs.

**Ageing and Healthcare Utilisation**

The COVID-19 pandemic demonstrated the vulnerability of older adults through the number of excess deaths across the globe and, in many countries, has resulted in uncertainty about the ways that healthcare can build resilience and promote health in this population. Globally, the diversity of social, cultural, and economic determinants means that there is a need to re-imagine utilisation and orientate services to build resilience, co-design health promotion initiatives, and focus on environmental protection and sustainability, especially in the light of climate change. There is an argument that age is the key determinant of health and that access to healthcare disproportionally affects older adult populations. Across one’s lifespan, patterns of health and wellbeing change (i.e., reduced mobility and increased risk of depression), and the demand for healthcare increases. Ageing, as a process, is universally well recognised and experienced by all, but multi-morbidity, cognitive frailty, functional incapacity, and reduced participation differentiate older adult populations. Across the world, the whole population is ageing in their national and local contexts, habitats, networks, communities, and households. Consumer economic status, personal resilience, and the individual or household ability to stay well (or well enough) enable older people to access healthcare. 

In theory, the supply of services should meet need-based demand, resources permitting, with no excess supply [11]. However, there are important considerations about the way healthcare is offered and accepted, particularly for older adults. Medical assessment, for example, is partially focused on the individual, based on their presentation of symptoms in the service of diagnostic acuity. Increasingly, multi-disciplinary teams, consisting of specialists in functional wellbeing, social factors, and psychological and pharmaceutical needs, are influencing the care package and enabling a wider service offer. The legitimate goal is to enable an older individual to self-manage and to share their perceptions of their health and begin to identify ‘what matters’ in relation to recovery or adaptation to residual health-related problems. This form of patient-centredness is a goal in relation to healthcare utilisation [3], with some suggesting that patient enablement reduces health service demand [12].

The Picker Institute’s principles of person-centred care [13] capture the priorities of patients; timely access and continuity of care are key facets of person-centred care, and these process improvements are a priority for those who are familiar with re-designing western medicine and traditional modes of care. Consistent focus on equalising access is, however, quite separate from notions of patient choice that are also often associated with healthcare utilisation. Choice should be understood as consumerist notion, based on the assumption that people, know, care, and can distinguish between the help they seek and receive. For those who fail to access services until they are either very old or very disabled and vulnerable, there are few choices other than acute care in a hospital or social care in residential facilities. Most healthcare systems would acknowledge that they need to address multiple structural challenges to achieve the inclusive ageing goal of the WHO [14], for elders to be enabled to live independently in their own homes for the longest time possible.

In a recent UN report [15], produced at the mid-way point towards the achievement targets outlined in the Sustainable Development Goals (SDG’s) of 2030, it was acknowledged that nearly half of the targeted activity towards sustainable environments and communities is off course. These targets, intended to create international agreement to combat climate change are significant in relation to population health with goals relating to air quality, poverty and biodiversity, each of which relates in some way to planetary health. Failure to meet SDGs disproportionally affects older people. The report refers to 575 million people globally who will be living in extreme poverty and nearly 2 billion who will have no access to clean cooking. Those who are older, vulnerable, and have multiple needs are at the eye of the storm in relation to climate risks and shock events. They represent the ‘burden’ of longevity, particularly when, due to poverty and ill health (non-communicable disease in OECD countries and communicable diseases in low- and middle-income countries), they have multiple needs for health promotion and reactive services. Importantly, the problem is often that the health infrastructure is too weak to meet these needs and/or too orientated towards reactive pharmacological interventions. 

Whilst increased demand is associated with ageing, it is more appropriately associated with healthcare utilisation and the services being appropriately designed to manage the multiple health risks in older age. Frailty [16] is a term imbued with a negative view about the capacity to recover and the older person’s capacity to retain their functional wellbeing but this individual focus denies society’s responsibility to provide protection and support for ageing people in their environments. People who are frail are disadvantaged by their state of ‘precarity’ in relation to daily life, often living in communities that demonstrate ageism and exclusion. Precarity is a state of discontinuity [17], where small changes can disrupt quality and safety and create uncertainty about the ability to cope independently, e.g., an accidental fall that results in a broken hip and propels an older adult into patient hood. Most healthcare services are busy treating the outcomes of this ‘precarity’ but carry out few initiatives to create safer and more inclusive environments that prevent loneliness and frailty. The term ‘precarity’ nicely captures the intersection of the personal health challenges experienced in the social and environmental context and the ability of the health infrastructure to supply services that meet the espoused demand. Accessible buses, well-insulated homes, reductions in air pollution, and social cohesion are all examples of how the physical environment profoundly prevents disability and enables older people to maintain their participation in communities [18].

Healthcare that aligns with the WHO’s healthy ageing policies could be remarkably beneficial to ameliorate the environmental risks. By endorsing a place-based health focus and incorporating multidomain interventions, communities would benefit from integrated healthcare to improve their quality of life [19]. The ability of households and communities to play crucial roles in self-managing their health (including the effects of climate change) could substantially benefit other generations as well, and by providing universal prevention strategies, people on lower incomes and the less healthy are not stigmatised. To address the dramatic and known inequity associated with access to health and care services internationally, there is a need to understand where low-income and old people live and begin a process of assessing health impacts. Strengthening the integration of the third sector [20] and using standardised protocols to engage the older population and prevent de-conditioning could be at the heart of value-based care and healthy ageing, and transform the healthcare system.
